# Differentiation of Human Pluripotent Stem Cells into Nephron Progenitor Cells in a Serum and Feeder Free System

**DOI:** 10.1371/journal.pone.0094888

**Published:** 2014-04-11

**Authors:** Minyong Kang, Yong-Mahn Han

**Affiliations:** 1 Graduate Schools of Medical Science and Engineering, KAIST, Daejeon, Republic of Korea; 2 Department of Biological Sciences, KAIST, Daejeon, Republic of Korea; Center for Molecular Biotechnology, Italy

## Abstract

**Objectives:**

Kidney disease is emerging as a critical medical problem worldwide. Because of limited treatment options for the damaged kidney, stem cell treatment is becoming an alternative therapeutic approach. Of many possible human stem cell sources, pluripotent stem cells are most attractive due to their self-renewal and pluripotent capacity. However, little is known about the derivation of renal lineage cells from human pluripotent stem cells (hPSCs). In this study, we developed a novel protocol for differentiation of nephron progenitor cells (NPCs) from hPSCs in a serum- and feeder-free system.

**Materials and Methods:**

We designed step-wise protocols for differentiation of human pluripotent stem cells toward primitive streak, intermediate mesoderm and NPCs by recapitulating normal nephrogenesis. Expression of key marker genes was examined by RT-PCR, real time RT-PCR and immunocytochemistry. Each experiment was independently performed three times to confirm its reproducibility.

**Results:**

After modification of culture period and concentration of exogenous factors, hPSCs can differentiate into NPCs that markedly express specific marker genes such as *SIX2*, *GDNF*, *HOXD11*, *WT1* and *CITED1* in addition to *OSR1*, *PAX2*, *SALL1* and *EYA1*. Moreover, NPCs possess the potential of bidirectional differentiation into both renal tubular epithelial cells and glomerular podocytes in defined culture conditions. In particular, approximately 70% of SYN-positive cells were obtained from hPSC-derived NPCs after podocytes induction. NPCs can also form *in vitro* tubule-like structures in three dimensional culture systems.

**Conclusions:**

Our novel protocol for hPSCs differentiation into NPCs can be useful for producing alternative sources of cell replacement therapy and disease modeling for human kidney diseases.

## Introduction

The kidneys have crucial regulatory roles in the body, including control of fluid-electrolyte, acid-base and blood pressure, and the excretion of waste products. Kidneys rarely recover functions following irreversible damages [1], [2]. Like other common chronic diseases such as diabetes and hypertension that are associated with modern lifestyle, the prevalence of kidney disease is increasing worldwide [3]. Chronic kidney diseases that preserve the residual kidney function deteriorate into irreversible end-stage renal disease (ESRD) [4], [5]. Treatment options of ESRD are limited to dialysis or renal transplantation [6]. Because complications of long-term dialysis and shortage of donated organs are unsolved problems to the ESRD patients, chronic kidney disease is best treated properly before it progresses into ESRD [3]. Despite recent progress in renal medicine, recovery and de novo regeneration of kidneys are elusive [7]. Stem cell therapy is emerging as one of the new approaches for replenishing damaged renal tissues in the field of regenerative medicine [2], [8]–[11]. Based on the normal renal developmental steps, metanephros is the final step of nephrogenesis during gestation [12]–[14]. It consists of two structures derived from intermediate mesoderm, the ureteric bud (UB) and metanephric mesenchyme (MM) [14], [15]. While UB gives rise to non-nephron tissues such as collecting ducts and ureters, MM turns into all segments of nephrons and renal interstitium [13], [16]. Thus, MM cells are considered renal progenitor cells, cap mesenchymal cells (nephron progenitor) and metanephric stromal cells (stromal progenitor) [14]. To obtain nephron progenitor cells (NPCs) that are capable of regenerating nephron-consisting cells, two approaches have been tried. First, human renal progenitor (hRPCs) cells have been isolated from diverse regions of fetal and adult kidney [17]–[22]. hRPCs yielded significant therapeutic effects in mouse models of renal failure, including reduction of BUN/Creatinine levels and replacement of destroyed epithelial cells within nephrons [20], [22], [23]. Nonetheless, there are several limitations for clinical application of hRPCs, including ethical problems in the isolation of hRPCs from human donors, low isolation efficiency and incomplete culture system for expansion of hRPCs [24]. Second, as an alternative source of NPCs, human pluripotent stem cells (hPSCs) are highly attractive because they are able to differentiate into various specialized cell types. Unfortunately, differentiation of renal lineage cells have been performed primarily in mouse ESCs [25]–[29]. In the human systems, renal lineage cells expressing several developing kidney genes were differentiated after treatments with retinoic acid, activin A and BMP7 [30]. Poximal tubule-like cells were derived from hESCs in defined culture conditions with functional characterization [31]. In addition, a robust induction of intermediate mesoderm (IM) cells from hPSCs was accomplished with phenotypic characterization [32]. However, these cells were not kidney progenitor cells which have more therapeutic potential *in vivo* [21], [24]. Ureteric bud-committed kidney progenitor-like cells was recently derived from hPSCs *in vitro* and *in vivo* [33]. Nonetheless, differentiation of nephron-committed progenitor cells is needed for therapeutic usage aiming at damaged nephrons. In this study, we differentiated hPSCs into NPCs by step-wise approach in a serum- and feeder-free condition. These NPCs were able to turn into two cell types within nephrons, renal tubular epithelial-cells and glomerular podocytes *in vitro*.

## Materials and Methods

### Maintenance of human pluripotent stem cells

H9 human ES cell line (H9) and human induced pluripotent stem cell line derived from CRL-2097 fibroblasts (CRL-iPSCs) were cultured using techniques we developed in earlier research [34]. The ES medium consisted of DMEM/F12 mixed with 20% knockout serum replacement, 1% penicillin-streptomycin (PenStrep), 1% nonessential amino acids (NEAA), 2 mM L-glutamate, 0.1 mM β-mercaptoethanol and 10 ng/mL FGF2 (R&D Systems, Minneapolis, MN). All of these materials, except for the FGF2, were purchased from Invitrogen (Carlsbad, CA). Every five days, colonies of H9 and CRL-iPSCs were mechanically sliced into small pieces, detached from the culture dish after treatment with 10 mg/ml collagenase IV (Gibco, Carlsbad, CA), and then transferred on fresh MEF to maintain the pluripotency. For all experiments, hPSCs were used between passages 40 and 60 in this study.

### Derivation of nephron progenitor cells (NPCs) from hPSCs

We differentiated hPSCs into NPCs via a series of differentiations into stage-specific cell types of pluripotent, primitive streak (PS), intermediate mesoderm (IM) and NPCs. First, undifferentiated hESCs and hiPSCs were re-plated on Matrigel (1∶40 dilution, BD Biosciences, Bedford, MA) coated 4-well culture dishes and cultured in mTeSR medium (Stemcell Technologies, Vancouver, BC, Canada) supplemented with 10 ng/ml FGF2 for four days under the feeder-free system. To induce PS cells derived from hPSCs, we modified the initial step of cardiomyocyte differentiation protocol previously described [35]. hPSCs were cultured in the basal differentiation serum-free media (RPMI-1640 medium containing 2% B27, 2 mM L-glutamine, 1% PenStrep) supplemented with both 100 ng/ml Activin A and 100 ng/ml Wnt3a (R&D Systems) for one day. For the next two days, hESCs were treated with 20 ng/ml BMP4 (Peprotech, Rocky Hill, NJ) and 10 ng/ml FGF2 while hiPSCs were treated with 20 ng/ml BMP4 and 10 ng/ml FGF2. For intermediate mesoderm induction from primitive streak, BMP4 was replaced with 50 ng/ml BMP7 (Peprotech) in the same basal medium supplemented with both 10 µM retinoic acid (RA; R&D Systems) and 10 ng/ml FGF2. In particular, hESCs were treated for six days while hiPSCs were treated for eight days with RA, BMP7 and FGF2 at this stage. In a final step for NPCs derivation, hPSC-derived IM cells were cultured in the medium supplemented with 150 ng/ml BMP7 and 50 ng/ml FGF2 without RA for 15 days. During differentiation into NPCs, basal culture medium was refreshed every two days.

### Differentiation of NPCs into renal epithelial cells consisting of nephrons

hPSC-derived NPCs were re-plated on fibronectin-coated dishes and stabilized in the same differentiation medium containing BMP7 (150 ng/ml) and FGF2 (50 ng/ml) for one day. They were incubated in renal epithelial cell growth medium (REGM™; Lonza, Allendale, NJ) supplemented with 50 ng/ml HGF (Peprotech) to develop into renal tubular epithelial cells (RTECs) for 21 days. For differentiation into glomerular podocytes, NPCs were cultured in VRAD medium, which consisted of DMEM/F12, 100 nM Vitamin D3 (Sigma-Aldrich, Saint Louis, Missouri), 100 µM RA and 10% FBS for seven days as previously described [19]. The medium was changed every two days during terminal differentiation.

### *In vitro* tubulogenic assay

For *in vitro* tubulogenic assay, hPSCs-derived NPCs were detached from culture dishes after treatment with Acutase (Innovative Cell Technologies, San Diego, CA) for ten minutes. Cells were filtered with 40 µm-filter mesh and centrifuged at 300×g for three minutes. Cell pellets were suspended in the renal epithelial cell growth medium (REGM™) at a concentration of 2×10^5^ cells/ml. The cell suspension was mixed with an equal volume of 200 µl collagen type I (BD biosciences) on ice for three-dimensional culture. After incubating collagen type I-cell mixtures on a 4-well dish at 37°C for 30 minutes for solidification of this mixture, 500 µl of REGM™ supplemented with 50 ng/ml HGF was gently added on the gels. Gels of collagen type I-cell mixtures were cultured in the REGM™ for 21 days. During cell culturing, the medium was changed every two days.

### RNA isolation and real-time RT PCR

Total RNA was extracted from hESCs and their derivatives using TRIzol (Invitrogen), followed by treatment with DNase I (Invitrogen). Total RNA (1 µg) was used to synthesize cDNA using Moloney murine leukemia virus (M-MLV) reverse transcriptase (Enzynomics, Seoul, Korea) with oligonucleotides (oligo-dT). To analyze the transcriptional expression of stage-specific genes, quantitative real time RT-PCR (q-PCR) was conducted on Bio-Rad CFX manager (Bio-Rad Laboratories, Hercules, CA) with SyberGreen. The reaction used the following protocol: 95 °C for 10 minutes followed by 40 cycles of 95 °C for 15 seconds (denaturation), 60 °C for 15 seconds (annealing) and 72 °C for 30 seconds (synthesis). Primers of target genes were designed by primer design tools in PubMed (http://www.ncbi.nlm.nih.gov/tools/primer-blast) and were adjusted at a final concentration of 0.5 pmole. All reactions were duplicated to exclude technical errors. For relative quantification, the expression level of target genes was normalized to that of glyceraldehyde-3-phosphate dehydrogenase (GAPDH) gene. Differences between the samples and the control were analyzed using the formula _ΔΔ_Ct. Relative expression of target genes was indicated as fold-changes compared with the control. All experiments for evaluating transcriptional expression by q-PCR were repeated three times for biological replication. Primers used in this study are summarized in Table S1.

### Immunocytochemistry

Samples were washed with PBS and fixed with 4% formaldehyde (Sigma-Aldrich) for 20 minutes at room temperature (RT). After washing three times with PBS containing 0.1% Tween 20 (PBST), 0.3% Triton X-100 (Sigma-Aldrich) was used to permeabilize the cell membrane. After washing three times with PBST, samples were blocked with 5% donkey serum (Jackson ImmunoResearch Laboratories, West Grove, PA) for one hour at RT and then incubated with primary antibodies at 4 °C overnight. After rinsing three times with PBST, samples were incubated with secondary antibody (donkey anti-mouse IgG-, donkey anti-goat IgG-, and donkey anti-rabbit IgG-Alexa 488 or 594; Invitrogen) for one hour. After washing six times with PBST, samples were mounted in VECTASHIELD Mounting Medium containing DAPI for five minutes. Immunolocalized cells were observed on a fluorescence microscope or Zeiss LSM 510 confocal microscope. Notably, following a previous report's methods [33], quantification of the number of cells expressing the key markers of specific differentiation stage was performed by manual counting in three randomly chosen fields. Primary antibodies used in this study are described in Table S2.

## Results

**Figure 1:**
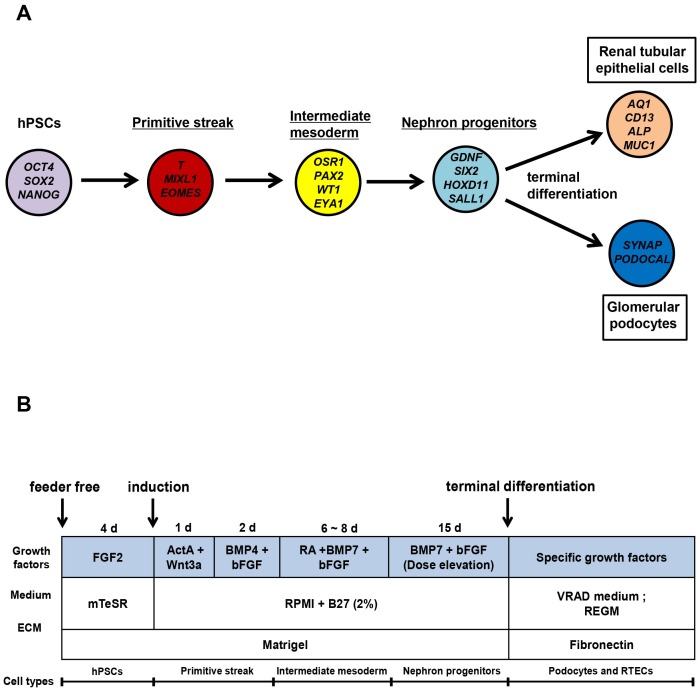
Overall protocol for differentiation of human pluripotent stem cells (hPSCs) into nephron progenitor cells (NPCs). A. Illustration of developmental stage-specific marker genes during differentiation of NPCs and NPC-derived fully differentiated renal epithelial cells. B. Detailed procedures for hPSCs differentiation into NPCs and its derivatives.

To establish optimal conditions for differentiation into NPCs, hPSCs were differentiated into stage-specific cell types of pluripotent, primitive streak (PS), intermediate mesoderm (IM) and nephron progenitor cells (NPCs) by treating with different combinations of growth factors. A schematic diagram and stage-specific marker genes during the entire differentiation phases are in Figure 1A. Detailed procedures for differentiation of NPCs and its bidirectional derivatives are in Figure 1B.

### Induction of hPSCs into primitive streak cells

**Figure 2:**
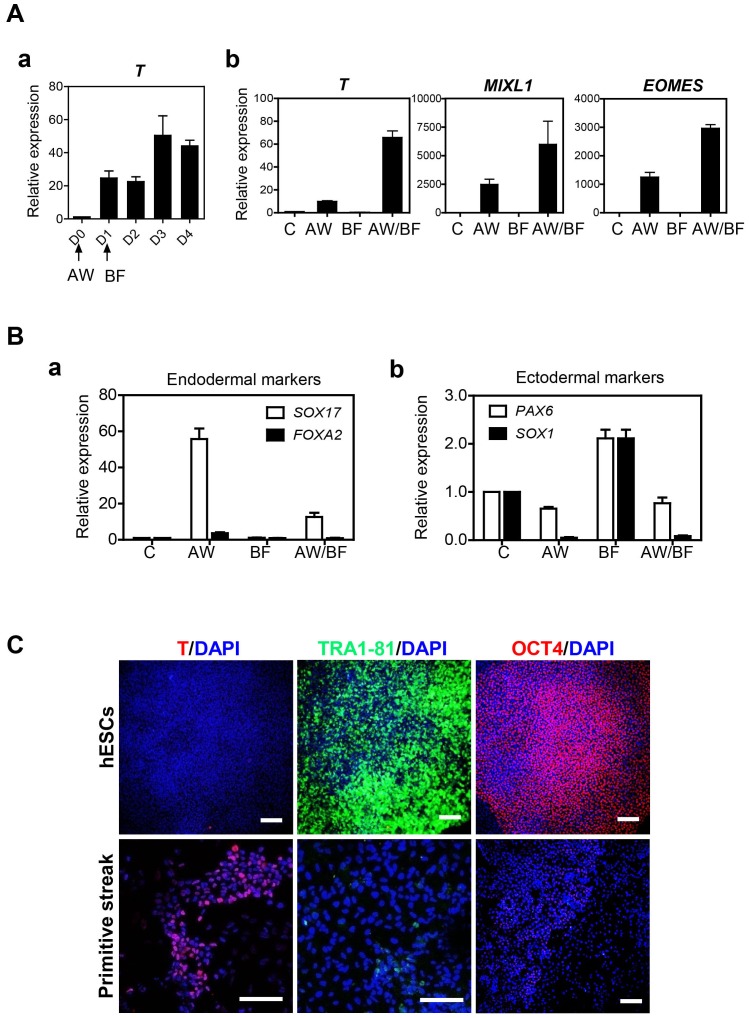
Induction of hESCs into primitive streak (PS) cells. A. (a) Optimal duration for PS induction was determined by transcription level of *T* compared with undifferentiated hESCs before treatments. Relative gene expression was normalized to GAPDH, and the values of fold-changes are represented by mean ± S.E.M (n = 3). (b) Comparison of expression levels of PS specific genes by combinatorial treatments with exogenous factors of Activin A, Wnt3a (AW) and BMP4, FGF2 (BF), in hESC-derived PS cells. Untreated samples were designated as (C). Relative transcriptional levels were normalized to GAPDH, and the bars show mean ± S.E.M (n = 3). B. (a) Determination of heterogeneity with other germ layers such as definitive endoderm (DE) and ectoderm by evaluating transcriptional expression of (a) DE and (b) ectoderm marker genes in hESC-derived PS cells. C. Immunofluorescence of T (red), TRA1-81 (green), and OCT4 (red) expression in the cells of induction day 0 (undifferentiated hESCs) and day 3 (primitive streak). Scale bars = 200 µm.

The initial stage of our protocol aimed at differentiating hPSCs toward PS population is based upon a reported method [35]. To determine optimal conditions for induction of hESCs to primitive streak cells, hESCs were incubated in medium supplemented with BMP4 and FGF2 (BF) for several days after treatments with Activin A and Wnt3a (AW) for one day. Transcriptional expression of *T* was enhanced at day 2 after addition of BMP4 and FGF2 (Figure 2A-a). Consecutive treatments with AW/BF showed higher transcription levels of PS genes such as *T*, *MIXL1*, and *EOMES* than treatments with two factors alone (AW or BF) (Figure 2A-b). However, AW/BF treatment did not up-regulate the transcription of other lineage marker genes such as *SOX17*, *FOXA2* (endoderm) and *PAX6*, *SOX1* (ectoderm) in hESCs-derivatives (Figure 2B-a, b). Immunostaining showed that consecutive treatments with AW/BF induced the expression of mesendodermal marker T and reduced the expression of pluripotent marker TRA1-81 and OCT4 in hESC-derived PS cells compared to undifferentiated hESCs (Figure 2C).

In hiPSCs, transcriptional expression of T was activated at day 3 after treatments with AW/BF (Figure S1A-a). In the same culture condition, the expression of T was enhanced while the expression of TRA1-81 was remarkably reduced in immunocytochemistry (Figure S1A-b). Higher concentration of BMP4 (50 ng/ml) was required to activate transcription of PS marker genes, but did not induce the transcriptional expression of endoderm and ectodermal genes (Figure S1A-c, d).

### Specification of intermediate mesoderm from PS cells

**Figure 3:**
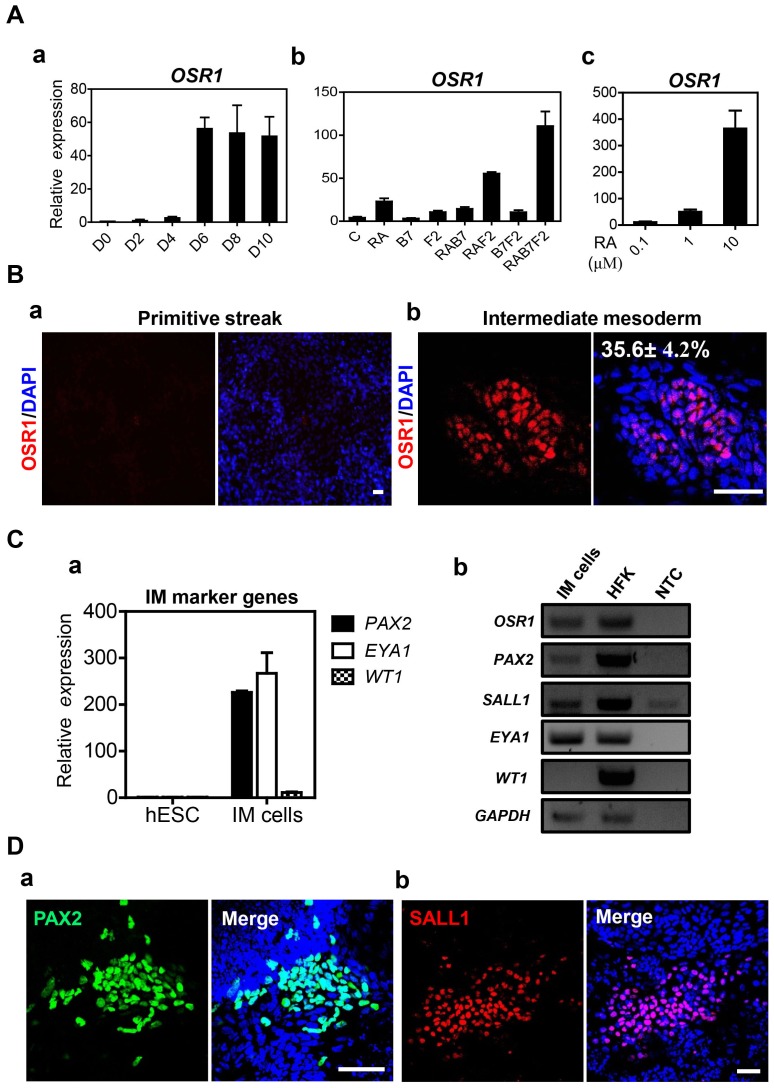
Specification of intermediate mesoderm (IM) from the hESC-derived PS cells. A. (a) Optimal timing for IM induction was determined by transcriptional expression level of *OSR1*, comparing with expression values of undifferentiated hESCs, in hESC-derived cells. Relative gene expression was normalized to GAPDH, and the values of fold-changes are represented by mean ± S.E.M (n = 3). (b) Comparison of *OSR1* transcripts by combinatorial treatments with exogenous growth factors of retinoic acid (RA), BMP7 (B7) and FGF2 (F2). Untreated samples were designated as (C). Transcriptional expression levels were normalized to GAPDH, and results of fold-change are indicated as mean ± S.E.M (n = 3). (c) Comparison of *OSR1* transcription levels by the concentration of RA treatment, including 0.1, 1 and 10 µM. Relative gene expression was normalized to GAPDH. The values of fold-changes are represented by mean ± S.E.M (n = 3). B. Immunocytochemistry for representative expression of OSR1 (red) in (a) hESC-derived PS and (b) IM cells. Quantification of the number of cells expressing the key markers of specific differentiation stage was performed by manual counting in three randomly chosen fields. Scale bars = 50 µm. C. Transcriptional expression of various IM marker genes were evaluated by (a) real time RT-PCR and (b) RT-PCR in hESC-derived IM cells. Human fetal kidney (HFK) cDNA was used as positive control. Relative transcriptional levels were normalized to GAPDH, and the bars show mean ± S.E.M (n = 3). D. Immunostaining of PAX2 (green) and SALL1 (red) at the end of IM induction in hESC-derivatives. Scale bars = 100 µm.

Intermediate mesoderm (IM) specified from PS stage is the most important step toward renal lineage differentiation [14]. Initially, the expression level of *OSR1*, a key upstream molecule in the renal development [15], was analyzed during IM cells differentiation. When hESC-derived PS cells were cultured in the basal medium supplemented with retinoic acid (RA), BMP7 (B7) and FGF2 (F2) for several days, the transcriptional level of *OSR1* was enhanced at day 6 after the treatment (Figure 3A-a). Combined treatment with RAB7F2 was more effective on transcriptional activation of *OSR1* than other treatment groups (Figure 3A-b). Of note, RA treatment resulted in the transcriptional activation of *OSR1* gene in a dose-dependent manner when comparing three different concentrations of RA, 0.1, 1, and 10 µM (Figure 3A-c). While hESC-derived PS cells did not express OSR1 (Figure 3B-a), the proportion of OSR1-positive cells was approximately 35% in hESC-derived IM cells in immunocytochemistry (Figure 3B-b). Other IM marker genes such as *PAX2*, *SALL1* and *EYA1* were detected at the transcription levels (Figure 3C). Interestingly, hESC-derived IM cells did not express *WT1* when compared to human fetal kidney (HFK) (Figure 3C-b). Expression of PAX2 and SALL1 were also detected in hESC-derived IM cells in immunocytochemistry (Figure 3D-a, b).

In hiPSCs, transcriptional expression of *OSR1* was enhanced at day 8 after treatments with RAB7F2 (Figure S1B-a). Similar to hESCs, RA treatment effectively activated *OSR1* gene expression in a dose dependent manner (Figure S1B-b). Other IM marker gene *PAX2* was also transcriptionally activated in hiPSC-derived IM cells (Figure S1B-c). Immunocytochemistry showed remarkable expressions of IM markers, including OSR1, PAX2, and SALL1 in hiPSC-derived IM cells (Figure S1B-d). Of note, approximately 25% of cells were positive for OSR1 after IM induction (Figure S1B-d).

### Differentiation of IM cells into NPCs and its characterization

**Figure 4:**
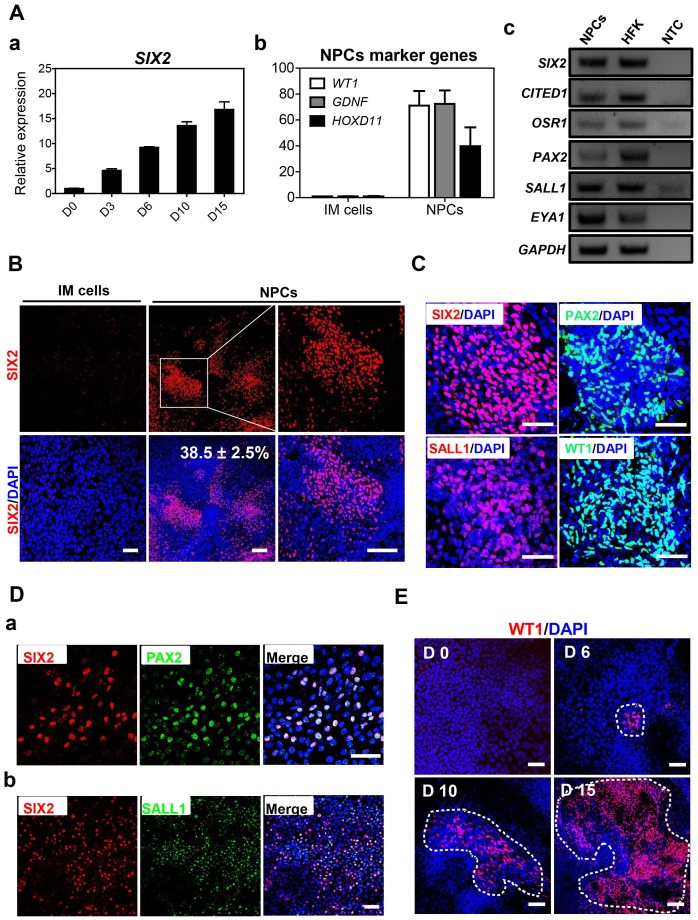
Differentiation of hESC-derived IM cells into NPCs. A. (a) Time course of the transcriptional expression of key NPCs marker *SIX2* in hESC-derived NPCs. Appropriate duration of NPCs induction was determined by relatively higher transcriptional expression of *SIX2* than other days. Transcription of other NPCs marker genes were examined by (b) real time RT-PCR and (c) RT-PCR in hESC-derived NPCs. Human fetal kidney (HFK) cDNA was used as positive control. Relative transcriptional levels were normalized to GAPDH, and the bars show mean ± S.E.M (n = 3). B. SIX2 expression rate was compared between hESC-derived IM cells and NPCs. Quantification of the number of cells expressing the key markers of specific differentiation stage was performed by manual counting in three randomly chosen fields. Scale bars = 200 µm. C. Several NPCs markers, including SIX2 (red), PAX2 (green), SALL1 (red) and WT1 (green) were notably detected in hESC-derived NPCs. Scale bars = 100 µm. D. Co-expression of other NPCs markers such as PAX2 (green) and SALL1 (green) with SIX2 (red) in hESC-derived NPCs in immunofluorescence. Scale bars = 100 µm. E. Immunocytochemistry for WT1 expression during NPCs induction in hESC-derivatives. WT1 expression (red) was evaluated at four different time points of NPCs induction (D0, D6, D10 and D15 of NPCs induction after IM stage). WT1-positive regions are designated by white lines. Scale bars = 100 µm.

For derivation of NPCs, IM cells were cultured in the basal medium containing high doses of BMP7 (150 ng/ml) and FGF2 (50 ng/ml) without RA. Transcripts of a key NPC marker gene *SIX2* was gradually up-regulated after day 3 of high doses BMP7/F2 treatments in hESC-derivatives (Figure 4A-a). Transcriptional expressions of other marker genes such as *GDNF*, *HOXD11* and *WT1* in addition to *CITED1*, *OSR1*, *PAX2*, *SALL1* and *EYA1* were enhanced in hESC-derived NPCs (Figure 4A-b, c). Immunocytochemistry showed that approximately 38% of SIX2-positive cells were obtained in hESC-derivatives after NPCs induction (Figure 4B). Other NPCs markers PAX2, SALL1 and WT1 were also markedly detected in hESC-derived NPCs (Figure 4C). PAX2 and SALL1 were co-expressed with SIX2 in hESC-derived NPCs (Figure 4D-a, b). Notably, WT1-expressing cells were appeared at day 6 of NPCs induction and the proportion of these cells increased in a time-dependent manner (Figure 4E).

**Figure 5:**
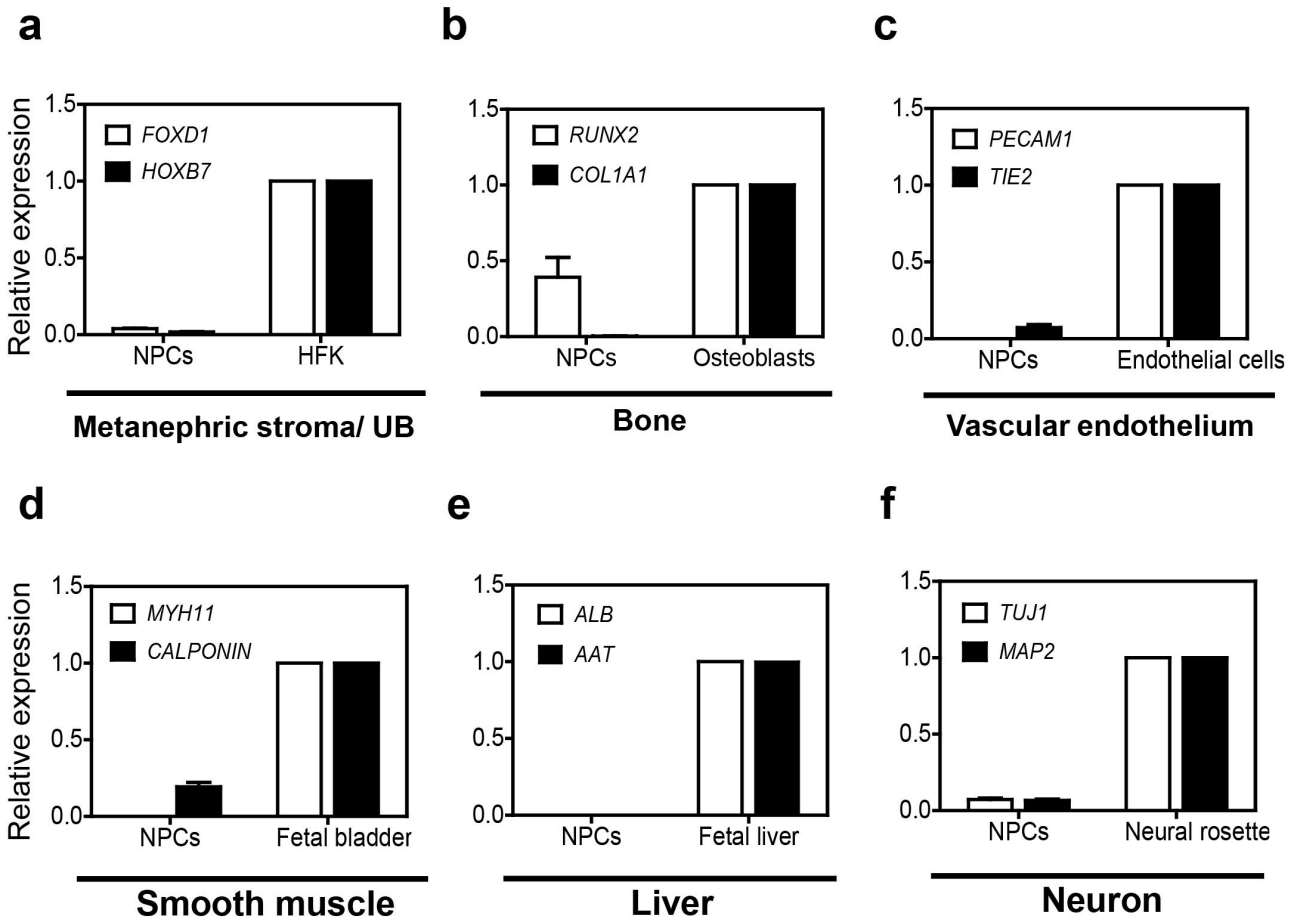
Evaluation of transcriptional activation of other inducible lineages markers in hESC-derived NPCs. Transcriptional expression of (a) *FOXD1* and *HOXB11* (metanephric stroma/ureteric bud), (b) bone (*RUNX2* and *COL1A1*), (c) vascular endothelium (*PECAM1* and *TIE2*), (d) smooth muscle (*MYOSIN11* and *CALPONIN*), (e) liver (*ALB* and *AAT*), and (f) neuron (*TUJ1* and *MAP2*) were analyzed by q-PCR in hESC-derived NPCs. Relative expression values were normalized to GAPDH, and fold-changes are shown by mean ± S.E.M (n = 3).

To determine whether hESC-derived NPCs primed into other lineages cells, we evaluated marker genes expression of common derivatives from three lineages in transcription levels. Transcripts of several marker genes of other lineages, including metanephric stroma/ureteric bud (*FOXD1* and *HOXB7*), bone (*RUNX2* and *COL1A1*), vascular endothelium (*PECAM1* and *TIE2*), smooth muscle (*MYH11* and *CALPONIN*), liver (*ALB* and *AAT*), and neuron (*TUJ1* and *MAP2*) were not detectable in hESC-derived NPCs (Figure 5). These results indicate that our differentiation protocol is suitable to induce NPCs without contamination with other lineages cells derived from definitive endoderm, mesoderm and ectoderm.

Similar to hESCs, several NPC-markers such as SIX2, PAX2, SALL1 and WT1 were notably expressed in hiPSC-derivatives (Figure S1C). The proportion of SIX2-positive cells was approximately 30% in hiPSC-derivatives (Figure S1C-b). Co-expression of PAX2 and SIX2 were also observed in these NPCs (Figure S1C-c).

### Differentiation potential of hPSC-derived NPCs toward renal tubular cells and glomerular podocytes

**Figure 6:**
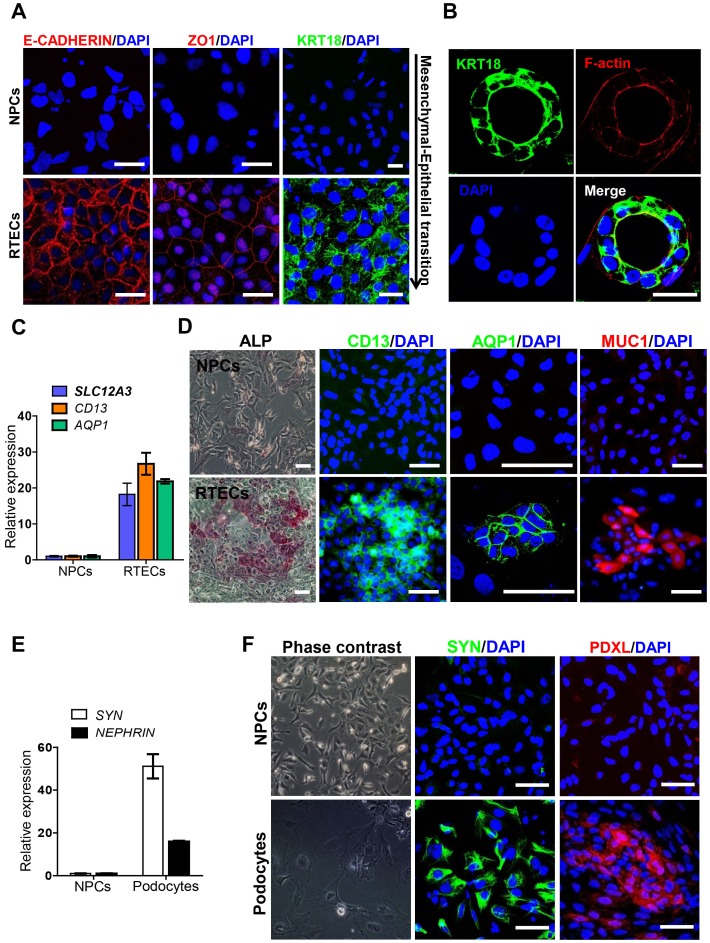
Differentiation potential of hESC-derived NPCs into fully differentiated cells within nephrons. A. Expression of epithelial markers such as E-CADHERIN, ZO1 and KRT18 in hESC-derived renal tubular epithelial cells (RTECs) compared with un-induced NPCs in immunocytochemistry, representing mesenchymal-epithelial transition. Scale bars = 50 µm. B. *In vitro* tubulogenesis of hESC-derived NPCs, cultured as three dimensional system mixed with collagen type I in renal epithelial cells growth medium (REGM™) for 21 days, which were analyzed by immunofluorescence with an epithelial marker KRT18 (green) and F-actin (red) for showing apicobasal polarization of RTECs in tubular structures. Scale bars = 50 µm. C. Transcriptional expression of RTEC-specific genes (*SLC12A3*, *CD13* and *AQP1*) was examined by real time RT-PCR. Relative expression levels were normalized to GAPDH, and the bars show mean ± S.E.M (n = 3). D. Expression of proximal renal tubular markers (ALP, alkaline phosphatase; AQP1, aquaporin1; CD13) and distal renal tubular marker MUC1 in hESC-derived RTECs compared with un-induced NPCs in immunocytochemistry. Scale bars = 100 µm. E. Transcriptional expression of podocyte-specific genes (*SYN* and *NEPHRIN*) was evaluated by real time RT-PCR. Relative expression levels were normalized to GAPDH, and the bars show mean ± S.E.M (n = 3). F. Phase contrast image and immunocytochemistry of podocyte-specific markers such as SYN (green) and PDXL (red) in hESC-derived glomerular podocytes compared with un-induced NPCs. Scale bars = 100 µm.

In defined culture condition following previously reported protocols [19], hPSC-derived NPCs were able to differentiate into renal tubular epithelial cell (RTECs) and glomerular podocytes. After RTEC induction for 21 days, hESC-derivatives expressed E-CADHERIN, ZO1 and KRT18 (Figure 6A), representing mesenchymal-epithelial transition (MET). NPCs also formed tube-like structures in three dimensional culture condition (Figure 6B). Tube-like structures were stained with an epithelium-specific maker KRT18 as well as F-actin representing apicobasal polarization of RTECs within tubular structures (Figure 6B). These RTECs showed transcriptional activation of RTEC marker genes, *SLC12A3*, *CD13* and *AQP1*, whereas un-induced NPCs did not express these genes (Figure 6C). Additionally, RTECs expressed makers of proximal (ALP, AQP1 and CD13) and distal tubular cells (MUC1), while un-induced NPCs did not express RTEC markers in immunocytochemistry (Figure 6D). After podocytes induction, hESC-derivatives expressed podocyte-specific genes *SYN* and *NEPHRIN* in transcription levels (Figure 6E) as well as SYN and PDXL in protein levels, compared to un-induced NPCs (Figure 6F). Of note, these cells showed aborized morphology on high magnification view (Figure 6F). In addition, approximately 70% of SYN-expressing cells were obtained in hESC-derivatives after podocytes induction.

In hiPSCs, markers of MET (E-CADHERIN, ZO1 and KRT18) and RTECs (ALP, CD13 and AQP1) were observed after 21 days of RTEC induction in immunocytochemistry (Figure S2A and S2B). These NPCs formed KRT18-positive tubule-like structures with apicobasal polarization in three dimensional cultures (Figure S2C). In addition, hiPSC-derivatives expressed podocyte-specific markers SYN and PDXL after podocytes induction (Figure S2D). Similar to hESCs, the proportion of SYN-positive cells was about at 70% in hiPSC-derivatives.

## Discussion

In contrast to other solid organs, kidneys consist of more than 10 specialized cell types in complex organization as functional units [11]. Thus, establishment of multipotent nephron progenitor cells (NPCs) is the most appropriate strategy to regenerate damaged renal cells within nephrons because progenitor cells can give rise to any cell types after administration into damaged tissues [9], [10], [21], [36]. Unfortunately, differentiation protocols of hPSCs into renal lineage-progenitor cells remain undefined. To our knowledge, this is the first report describing *in vitro* differentiation of hPSCs into NPCs in a chemically defined serum- and feeder-free system via developmental stage-dependent manner.

In normal kidney developmental processes, metanephric mesenchyme (MM) contain a population of renal progenitor cells expressing specific transcription factors, including *SIX2*, *SALL1*, *CITED1*, *GDNF* and *HOXD11* analogues in addition to *OSR1*, *PAX2*, *EYA1*, *WT1* that continuously appear following the IM stage [12], [16], [24], [37]. Because mutations of these genes resulted in failure of proper nephrogenesis [38]–[42], the expression of these transcription factors is critical for efficient induction of NPCs from hPSCs in our protocol. Interestingly, although WT1 is initially expressed in IM stage and persistently expressed during MM induction as a key transcription factor for *in vivo* nephrogenesis [43], [44], WT1 did not appear at the end of IM induction in this study. Instead, transcriptional activation of *WT1* and the proportion of WT1-positive cells in immunocytochemistry were increased after NPCs induction. These results indicate that there are temporal differences in the expression pattern of WT1 during renal lineage differentiation between *in vitro* and *in vivo*.

To differentiate hPSCs into NPCs through the consecutive developmental stages of PS and IM, different combinations of growth factors were optimized in this study. BMP7 is a transcription factor for IM and metanephric mesenchymal (MM) specification from the early stage of mesoderm [37]. Thus, BMP7 was used as a key exogenous factor during induction of IM cells from PS. We believe that RA is also needed for IM differentiation because it is secreted from the paraxial mesoderm and acts as an important signaling factor for specification of primitive streak into IM [25], [27], [30], [45], [46]. In contrast to the recent study suggesting that retinoic acid (RA) is not needed to induce OSR1-expressing IM cells [32], RA addition was highly effective in inducing IM in a dose dependent manner in this study. There are several factors that may lead to these different outcomes. First, Mae *et al.* used a low concentration of RA (0.1 µM), whereas we used a higher concentration of RA (10 µM) that was more effective on IM induction compared to 0.1 and 1 µM RA. Second, they used a different growth factors combination (100 ng/ml BMP7+3 µM CHIR99021+0.1 µM RA) from that which we used (150 ng/ml BMP7+10 ng/ml FGF2+10 µM RA) during IM induction. Finally, they used cell lines (a hiPSC line, 253G1; a hESC line, khES3) different from that which we used (a hiPSC line, CRL-2097; a hESC line, H9). We presume that these differences contribute to the differing the effect of RA on IM induction from hPSCs.

For NPCs induction, high doses of BMP7 (150 ng/ml) and FGF2 (50 ng/ml) were added for the specification of NPCs from IM cells because ureteric bud-secreting factors BMP7 and FGF2 have been known as the most important signaling factors to proliferate and sustain self-renewal potential of renal progenitor cells in MM [12], [16]. Notably, hiPSCs showed relatively lower efficiency of IM and NPCs differentiation compared to hESCs. These discrepancy may be attributed to epigenetic memory and alteration of iPSCs, making some iPSCs more resistant to exogenous signaling cues for differentiation into specific lineages [47].

There are several limitations in this study. First, our results showed only about 35% induction efficiency for SIX2-positive NPCs. However, a specific isolation of NPCs population with higher efficiency will be needed when considering clinical applications. To achieve this, we can examine the effects of many other growth factors on hPSC differentiation into NPCs by using high-throughput screening system and finding optimal combinations of NPC-inducing factors. Alternatively, we can identify the ideal markers to recover hPSC-derived NPCs to improve their purity. Second, we conducted only *in vitro* experiments that do not fully recapitulate *in vivo* conditions. To reduce the unknown hazards from implanted cells, *ex vivo* and *in vivo* experiments are needed to confirm that hPSC-derived NPCs can integrate into kidney structures without serious problems such as teratoma formation. In addition, evaluating the clinical efficacy of hPSC-derived NPCs will require *in vivo* renal failure models. Such additional experiments offer the potential of expanding knowledge in the field of regenerative medicine.

## Conclusions

In summary, we differentiated hPSCs into NPCs that were further specified into RTECs and podocytes in a serum- and feeder-free system. This protocol can be useful for generating alternative sources of cell therapy as well as *in vitro* platform for the investigation of human kidney diseases at the molecular and cellular levels.

## Supporting Information

Figure S1 **Induction of hiPSCs into NPCs.** A. Induction of PS cells. (a) Optimal timing of transcriptional expression of *T* during PS cells induction from hiPSCs by AW/BF serial treatments. Undifferentiated hiPSCs were used as control. Relative gene expression was normalized to GAPDH and fold-change values are represented by mean ± S.E.M (n = 3). (b) Immunostaining of T (red) and TRA1-81 (green) expression in hiPSC-derived PS cells. Scale bars = 200 µm. Comparison of transcriptional activation of (c) PS marker genes and (d) other lineage markers, including *SOX17* (definitive endoderm) and *PAX6* (ectoderm), between different concentrations of BMP4 (0, 20 and 50 ng/ml). B. Specification of IM cells. (a) Appropriate time point of IM-induction was determined by transcriptional activation levels of *OSR1* in hiPSC-derivatives. Relative gene expression was normalized to GAPDH, and fold-changes are represented by mean ± S.E.M (n = 3), compared with expression values of undifferentiated hiPSCs. (b) Comparison of *OSR1* expression levels by concentration of retinoic acid (RA) treatment, including 0.1, 1 and 10 µM in hiPSC-derived cells. Untreated samples were used as control. Relative gene expression was normalized to GAPDH. The values of fold-changes are shown by mean ± S.E.M (n = 3). (c) Transcriptional expression of several IM marker genes such as *OSR1* and *PAX2* were evaluated by real time RT-PCR in hiPSCs-derived IM cells. No factors indicate un-treated control. Relative gene expression was normalized to GAPDH, and fold-changes are represented by represented by mean ± S.E.M (n = 3). (d) Immunocytochemistry for the expression of key IM markers, including OSR1 (red), PAX2 (green) and SALL1 (red) in hiPSC-derived IM cells. Scale bars = 100 µm. C. Immunocytochemistry analysis for SIX2 expression in (a) hiPSC-derived IM cells and (b) NPCs. Quantification of the number of cells expressing the key markers of specific differentiation stage was performed by manual counting in three randomly chosen fields. Scale bars = 100 µm. (c) Immunocytochemistry of other NPCs markers such as PAX2 (green), SALL1 (red) and WT1 (green). Scale bars = 100 µm.
(TIF)

Figure S2 **Differentiation of hiPSC-derived NPCs into fully differentiated nephron-consisting cells.** A. Evaluation of epithelial markers expression, including E-CADHERIN, ZO1 and KRT18 in hiPSC-derived RTECs by immunocytochemistry, indicating mesenchymal-epithelial transition. Scale bars = 50 µm. B. Expression of proximal renal tubular markers (ALP, AQP1 and CD13) and distal renal tubular marker MUC1 in hiPSC-derived RTECs in immunocytochemistry. Scale bars = 100 µm. C. Immunocytochemistry for KRT18 (green) and F-actin (red) of 3D tubule-like structures formed by culturing hiPSC-derived NPCs with collagen type I-cells mixtures in REGM™ for 21 days. Scale bars = 50 µm. D. Expression of glomerular podocyte-specific markers such as SYN (green) and PDXL (red) in hiPSC-derived glomerular podocytes in immunofluorescence. Scale bars = 100 µm.
(TIF)

Table S1 **The information of real time and RT-PCR primers.**
(DOCX)

Table S2 **The information of primary antibodies used for immunostaining.**
(DOCX)

Table S3 **Abbreviations used in this study.**
(DOCX)
