# Supplementary figures and images for: Differentiation of Human Pluripotent Stem Cells into Nephron Progenitor Cells in a Serum and Feeder Free System
Source: PLoS One. 2014 Apr 11;9(4):e94888. doi: 10.1371/journal.pone.0094888 (PMC3984279; doi:10.1371/journal.pone.0094888)

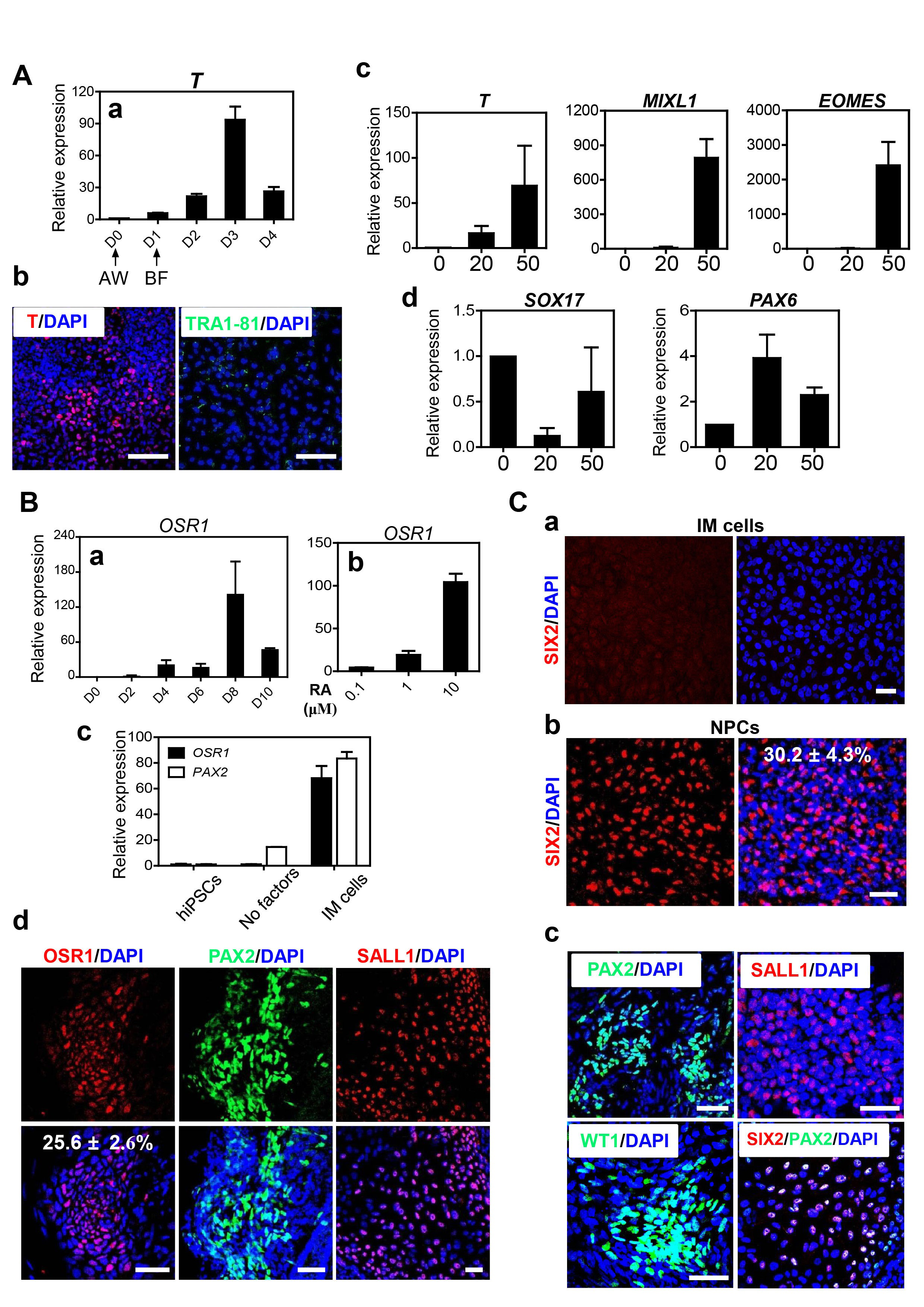

Supplement: Figure S1 — Induction of hiPSCs into NPCs. A. Induction of PS cells. (a) Optimal timing of transcriptional expression of T during PS cells induction from hiPSCs by AW/BF serial treatments. Undifferentiated hiPSCs were used as control. Relative gene expression was normalized to GAPDH and fold-change values are represented by mean ± S.E.M (n = 3). (b) Immunostaining of T (red) and TRA1-81 (green) expression in hiPSC-derived PS cells. Scale bars = 200 µm. Comparison of transcriptional activation of (c) PS marker genes and (d) other lineage markers, including SOX17 (definitive endoderm) and PAX6 (ectoderm), between different concentrations of BMP4 (0, 20 and 50 ng/ml). B. Specification of IM cells. (a) Appropriate time point of IM-induction was determined by transcriptional activation levels of OSR1 in hiPSC-derivatives. Relative gene expression was normalized to GAPDH, and fold-changes are represented by mean ± S.E.M (n = 3), compared with expression values of undifferentiated hiPSCs. (b) Comparison of OSR1 expression levels by concentration of retinoic acid (RA) treatment, including 0.1, 1 and 10 µM in hiPSC-derived cells. Untreated samples were used as control. Relative gene expression was normalized to GAPDH. The values of fold-changes are shown by mean ± S.E.M (n = 3). (c) Transcriptional expression of several IM marker genes such as OSR1 and PAX2 were evaluated by real time RT-PCR in hiPSCs-derived IM cells. No factors indicate un-treated control. Relative gene expression was normalized to GAPDH, and fold-changes are represented by represented by mean ± S.E.M (n = 3). (d) Immunocytochemistry for the expression of key IM markers, including OSR1 (red), PAX2 (green) and SALL1 (red) in hiPSC-derived IM cells. Scale bars = 100 µm. C. Immunocytochemistry analysis for SIX2 expression in (a) hiPSC-derived IM cells and (b) NPCs. Quantification of the number of cells expressing the key markers of specific differentiation stage was performed by manual counting in three randomly chosen f [file pone.0094888.s001.tif]

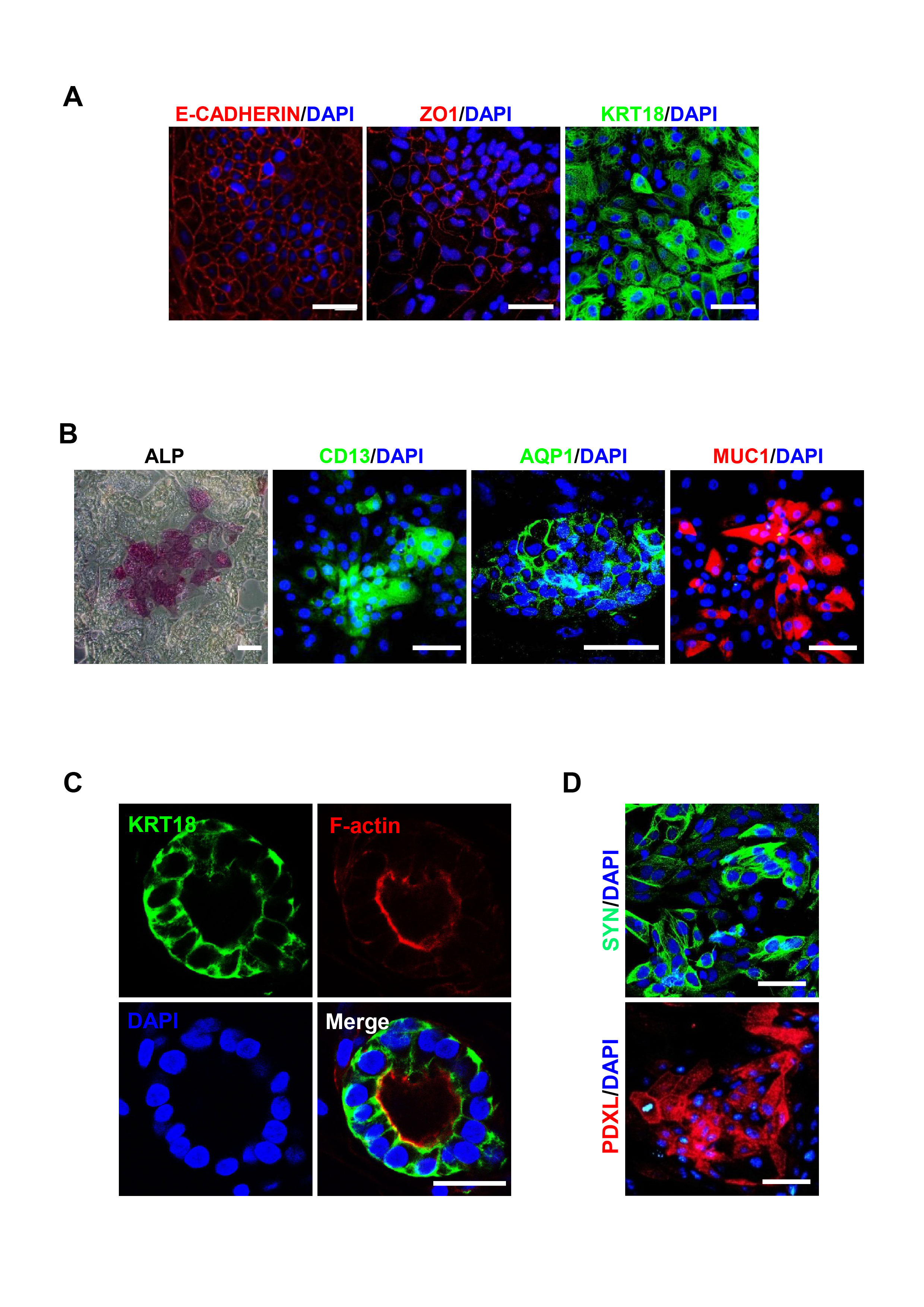

Supplement: Figure S2 — Differentiation of hiPSC-derived NPCs into fully differentiated nephron-consisting cells. A. Evaluation of epithelial markers expression, including E-CADHERIN, ZO1 and KRT18 in hiPSC-derived RTECs by immunocytochemistry, indicating mesenchymal-epithelial transition. Scale bars = 50 µm. B. Expression of proximal renal tubular markers (ALP, AQP1 and CD13) and distal renal tubular marker MUC1 in hiPSC-derived RTECs in immunocytochemistry. Scale bars = 100 µm. C. Immunocytochemistry for KRT18 (green) and F-actin (red) of 3D tubule-like structures formed by culturing hiPSC-derived NPCs with collagen type I-cells mixtures in REGM™ for 21 days. Scale bars = 50 µm. D. Expression of glomerular podocyte-specific markers such as SYN (green) and PDXL (red) in hiPSC-derived glomerular podocytes in immunofluorescence. Scale bars = 100 µm. (TIF) [file pone.0094888.s002.tif]
