# Supplementary material for: Differentiation of Human Pluripotent Stem Cells into Nephron Progenitor Cells in a Serum and Feeder Free System
Source: PLoS One. 2014 Apr 11;9(4):e94888. doi: 10.1371/journal.pone.0094888 (PMC3984279; doi:10.1371/journal.pone.0094888)
Supplement: Table S1 — The information of real time and RT-PCR primers. (DOCX) [file pone.0094888.s003.docx]

**Table S1.** The information of real time and RT-PCR primers

| **Gene** | **Accession number** | **Forward sequence** | **Reverse sequence** | **Product size (bp)** |
| --- | --- | --- | --- | --- |
| *GAPDH* | NM_001256799.1 | ctt cgc tct ctg ctc ctc ct | gtt aaa agc agc cct ggt ga | 152 |
| *T* | NM_031944 | cag tgg cag tct cag gtt aag aag ga | cgc tac tgc agg tgt gag caa | 122 |
| *MIXL1* | NM_003181 | tcc agg atc cag gta tgg tt | cgt ttc agt tcc agg agc ac | 123 |
| *EOMES* | NM_005442 | atg ctg aag agt ata gta aag aca | aac acc acc aag tcc atc | 128 |
| *SOX17* | NM_031439 | aaa gac cca ggg tac cta aa | agg aag aca aat tct cac agc ag | 255 |
| *FOXA2* | NM_021784 | ctg agc gag atc tac cag tgg a | agt cgt tga agg aga gcg agt | 103 |
| *PAX6* | NM_000280.3 | agc cca gta taa gcg gga gt | cta gcc agg ttg cga aga ac | 135 |
| *SOX1* | NM_005986.2 | cac aac tcg gag atc agc aa | ggt act tgt aat ccg ggt gc | 133 |
| *OSR1* | NM_145260 | cgg aga gtg agt gga gag | tga agc aga tac agg gat tac a | 122 |
| *PAX2* | NM_003987 | acg ccc att aaa gca cag | tta cag aga aag agc caa caa a | 149 |
| *SALL1* | NM_002968 | agc gaa gcc tca aca ttt cca atc c | aat tca aag aac tcg gca cag cac c | 147 |
| *EYA1* | NM_000503 | gat gtc aag tgt cag taa gga t | aag tga ggt ggt agg aga g | 133 |
| *WT1* | NM_000378 | tgt gtg ttg tgt tgt gtt t | tgt caa aga gca aat cat tat ca | 105 |
| *SIX2* | NM_016932 | ctt gcc acc gtt cat tct | gga cca gga cac aga gta | 103 |
| *GDNF* | NM_000514 | caa gaa gca gca gtt acc a | gag cag aaa gga cag aga ag | 89 |
| *HOXD11* | NM_021192.2 | tgg aac gcg agt ttt tct tt | ctg cag acg gtc tct gtt ca | 149 |
| *CITED1* | NM_001144886 | gct gct aat gcc aag tgt | gcc cgt ctc ttt gta agt aac | 90 |
| *FOXD1* | [NM_004472.2](http://www.ncbi.nlm.nih.gov/nuccore/NM_004472.2) | tgc ggg tcc ctc tat tta tg | taa cgc ctg gac ctg aga at | 190 |
| *HOXB7* | [NM_004502.](http://www.ncbi.nlm.nih.gov/nuccore/NM_004472.2)3 | gtg gac tgt ggg tct gga ct | gaa cac gcg agt ggt agg tt | 114 |
| *SCL12A3* | [NM_000339.2](http://www.ncbi.nlm.nih.gov/entrez/viewer.fcgi?db=nucleotide&id=186910314) | gga ctt tgt ggg cac ctt | ggc atc ctc tgc ttg tgg | 84 |
| *CD13* | [NM_001150.2](http://www.ncbi.nlm.nih.gov/entrez/viewer.fcgi?db=nucleotide&id=157266299) | atc tct tac gca cat cag g | tcc agt tct ctt cgt cgt a | 90 |
| *AQP1* | [NM_198098.2](http://www.ncbi.nlm.nih.gov/entrez/viewer.fcgi?db=nucleotide&id=297307114) | cct cct ggc tat tga cta ca | ggt tgc tga agt tgt gtg t | 86 |
| *SYN* | [NM_007286.5](http://www.ncbi.nlm.nih.gov/entrez/viewer.fcgi?db=nucleotide&id=261278294) | agc cca agg tga ccc cga at | ccc tgt cac gag gtg ctg gc | 162 |
| *NEPHRIN* | [NM_004646.3](http://www.ncbi.nlm.nih.gov/entrez/viewer.fcgi?db=nucleotide&id=260593642) | tgg tct aca gcc ttc tac a | ctt tgt cag cca gtc cac | 80 |
| *RUNX2* | NM_001024630.3 | gac agc ccc aac ttc ctg t | ccg gag ctc agc aga ata at | 159 |
| *COL1A1* | XM_005257059.1 | act tgc ttg aag acc cat gc | ggt gtt tga gca ttg cct tt | 196 |
| *PECAM1* | NM_000517 | tgc gaa tcg atc agt gga | acc ggg gct atc acc ttc | 209 |
| *TIE2* | XM_005251563.1 | cct tag tga cat tct tcc | gca aaa atg tcc acc tgg | 243 |
| *MYH11* | NM_002474.2 | cag ttc gaa agg gat ctc ca | gta gct gct tga tgg ctt cc | 220 |
| *CALPONIN* | NM_001299 | agg ctc cgt gaa gaa gat ca | cca cgt tca cct tgt ttc ct | 215 |
| *ALBUMIN* | NM_000477 | tgc aca gaa tcc ttg gtg aa | ttc acg agc tca aca agt gc | 173 |
| *AAT* | NM_000295 | gaa gtc aag gac acc gag ga | gct ggc aga cct tct gtc tt | 261 |
| *TUJ1* | NM_006086.3 | ggg cct ttg gac atc tct tc | cct ccg tgt agt gac cct tg | 90 |
| *MAP2* | XM_005246567.1 | gtg gcg gac gtg tga aaa ttg ag | ctg gat ctg cct ggg gac tgt g | 205 |
