# Supplementary material for: Differentiation of Human Pluripotent Stem Cells into Nephron Progenitor Cells in a Serum and Feeder Free System
Source: PLoS One. 2014 Apr 11;9(4):e94888. doi: 10.1371/journal.pone.0094888 (PMC3984279; doi:10.1371/journal.pone.0094888)
Supplement: Table S2 — The information of primary antibodies used for immunostaining. (DOCX) [file pone.0094888.s004.docx]

**Table S2.** The information of primary antibodies used for immunostaining

| **Antibody** | **Host** | **Dilution factor** | **Industry** |
| --- | --- | --- | --- |
| T | Goat | 1:200 | R&D Systems |
| TRA1-81 | Mouse | 1:100 | Millipore |
| OSR1 | Rabbit | 1:40 | Sigma-Aldrich |
| PAX2 | Rabbit | 1:200 | Abcam |
| SALL1 | Rabbit | 1:400 | Abcam |
| WT1 | Rabbit | 1:200 | Abcam |
| SIX2 | Mouse | 1:200 | Abnova |
| E-CADHERIN | Mouse | 1:50 | BD Pharmingen |
| ZO1 | Rabbit | 1:400 | Millipore |
| KRT18 | Mouse | 1:200 | Abcam |
| CD13 | Rabbit | 1:200 | Abcam |
| AQP1 | Rabbit | 1:300 | Abcam |
| MUC1 | Mouse | 1:200 | Abcam |
| SYNAPTOPODIN | Mouse | 1:200 | PROGEN Biotechnik |
| PODOCALYXIN (TRA1-60) | Mouse | 1:100 | Millipore |
