# Supplementary material for: Differentiation of Human Pluripotent Stem Cells into Nephron Progenitor Cells in a Serum and Feeder Free System
Source: PLoS One. 2014 Apr 11;9(4):e94888. doi: 10.1371/journal.pone.0094888 (PMC3984279; doi:10.1371/journal.pone.0094888)
Supplement: Table S3 — Abbreviations used in this study. (DOCX) [file pone.0094888.s005.docx]

**Table S3.** Abbreviations

| **Abbreviation** | **Description** |
| --- | --- |
| hPSCs | Human pluripotent stem cells |
| hESCs | Human embryonic stem cells |
| hiPSCs | Human induced pluripotent stem cells |
| PS | Primitive streak |
| DE | Definitive endoderm |
| IM | Intermediate mesoderm |
| NPCs | Nephron progenitor cells |
| MM | Metanephric mesenchyme |
| RTECs | Renal tubular epithelial cells |
| SYNAPTOPODIN | SYN |
| PODOCALYXIN | PDXL |
| AW | Activin and Wnt3a |
| BF | BMP4 and FGF2 |
| RA | Retinoic acid |
| B7 | BMP7 |
| F2 | FGF2 |
| REGM^TM^ | Renal epithelial cells growth medium |
